# The First Nationwide Mapping of Aesthetic Treatments in Medicine in Central Europe: A Descriptive Observational Analysis

**DOI:** 10.1007/s00266-026-05992-z

**Published:** 2026-07-07

**Authors:** Melanie Matousek, Reinhard Edelmoser, Susanna Steffan, Adrian Meißner, Jens Kleine, Christopher Migsich, Stefanie Nickl, Wolfgang Michlits

**Affiliations:** 1 Department of Plastic, Aesthetic and Reconstructive Surgery, University Hospital Wiener Neustadt-NOE LGA, Corvinusring 3-5, 2700 Wiener Neustadt, Lower Austria Austria; 2 Department of Medicine, Faculty of Medicine and Dentistry, Danube Private University, 3500 Krems, Austria

**Keywords:** Aesthetic medicine, Non-surgical aesthetic treatments, Central Europe, Aesthetic services, Aesthetic practitioners

## Abstract

**Background:**

Aesthetic medicine has become an integral part of modern lifestyle, with global procedure volumes rising by nearly 40% over the past 4 years. In several countries, non-physicians are legally allowed to perform non-surgical aesthetic treatments, reflecting a diversification of providers. In Austria, the legal framework is thoroughly regulated. Aesthetic treatments without medical indication may only be carried out by licensed physicians, and authorization is tied to specialty-specific competence. Despite this regulation, systematic data on providers of aesthetic treatments are lacking.

**Methods:**

A systematic internet-based analysis was performed on all websites of office-based physicians, regardless of their specialization, including both private and insurance-based practitioners listed by the Austrian Medical Chamber at this time. 19388 office-based physicians could be included. The study was designed as a descriptive observational analysis.

**Results:**

Among office-based physicians, 7.1% (n=1436) list aesthetic services on their practice websites. 62.5% (n=12566) show no reference, and 30.4% (n=6116) could not be evaluated due to the absence of a personal website. Among physicians offering non-surgical aesthetic treatments (78.9%; 1133/1436), dermatologists accounted for 32.7%, general practitioners (GPs) for 20.3%, and plastic surgeons for 18%. Botulinum toxin and dermal filler treatments were referenced by nearly all specialties.

**Conclusion:**

This first nationwide web analysis shows that most non-surgical aesthetic treatments are offered outside the field of plastic surgery, with dermatologists and GPs representing the leading provider groups. The online listing of botulinum toxin and filler treatments across nearly all specialties shows that many different medical disciplines are actively involved in aesthetic medicine in Central Europe.

- aesthetic services of all office-based physicians in Central Europe
- descriptive observational website-based analysis
- many different medical disciplines are actively involved in aesthetic services

**Level of Evidence IV:**

This journal requires that authors assign a level of evidence to each article. For a full description of these Evidence-Based Medicine ratings, please refer to the Table of Contents or the online Instructions to Authors www.springer.com/00266.

## Introduction

Aesthetic medicine has become an integral part of modern lifestyle. Global activity has risen markedly, with the International Society of Aesthetic Plastic Surgery (ISAPS) reporting a ~40% increase in total procedures over the past 4 years, continuing a decade-long upward trend [1, 2]. Growth has been driven largely by minimally invasive, non-surgical treatments [3]. Nevertheless, recent international surveys from ISAPS 2024 show a 4.8% global decrease in comparison to the results from 2023 [4]. The American Society of Plastic Surgeons (ASPS) statistics indicate stable volumes within member practices despite broader economic uncertainty and competition from non-physician providers [5, 6]. Aesthetic treatments are performed without medical indication and at patient request to modify aspects of appearance [7]. In several countries, non-physicians are legally permitted to deliver non-surgical aesthetic treatments. Non-surgical facial aesthetics (NSFA), particularly injectables such as botulinum toxin and dermal fillers, remain central due to short downtime, predictable risk profiles, and rapid effects [5]. Recent data, however, suggest divergent trends. According to ISAPS, global totals for non-surgical procedures declined by approximately 3.1% from 2023 to 2024, with injectable procedures showing a more pronounced decrease of 8.1% [4]. In contrast, ASPS statistics report a 4% increase in neuromodulator injections during the same period. These differences may partly reflect methodological variation; ASPS statistics include procedures performed not only by board-certified plastic surgeons but also by non-member dermatologists and otolaryngologists [5].

Beyond procedure counts reported in international surveys, little is known about the specialty-specific provider landscape of aesthetic medicine at the national level. In Austria, the ÄsthOpG (Federal Law BGBI. I No. 80/2012) provides the legal basis for aesthetic interventions without medical indication [8]. According to the Austrian Medical Chamber, non-surgical aesthetic treatments may in principle be performed only by licensed physicians. General practitioners and fully licensed physicians may conduct such treatments provided they have appropriate training, and no specialty-specific restrictions apply. Specialists may perform aesthetic procedures that fall within their respective fields of expertise [7, 8]. This framework aims to ensure patient safety while accommodating growing demand. Despite this regulation, systematic data on providers of aesthetic treatments in Austria are lacking, leaving the distribution across disciplines unclear. We therefore conducted for the first time a nationwide, internet-based descriptive analysis of office-based physicians advertising non-surgical aesthetic treatments to characterize provider distribution and address this data gap. Rather than estimating procedure numbers, this study maps publicly advertised aesthetic services across Austria and links them to medical specialties. To our knowledge, this is the first study in Central Europe to evaluate all office-based physicians by specialty and reported non-surgery aesthetic treatments.

## Materials and Methods

Between January and August 2025, we conducted a systematic internet-based search on all websites of office-based physicians in Austria regardless of their specialization, including both private and insurance-based practitioners in Austria who were officially listed by the Austrian Medical Chamber at this time. The study was designed as a descriptive observational analysis, focusing on the structure, medical specialties, and non-surgery aesthetic treatments offered by physicians with own practices. The internet was selected as the primary data source because it represents the most widely used and publicly accessible medium for information dissemination. Physicians without their own or without a functional website at the time of data collection were classified as not assessable (NA) with respect to aesthetic treatments. For these cases, only basic demographic data were recorded. Data were systematically retrieved from the official websites of the Austrian Medical Chamber and from the physicians’ individual practice homepages. Extracted variables included zip code, medical specialty, and the provision of aesthetic treatments. When aesthetic procedures were offered, details were recorded according to the Guidelines of Aesthetic Plastic Surgery of the Austrian Society for Plastic, Reconstructive, and Aesthetic Surgery [9]. Aesthetic treatments included botulinum toxin injections, dermal fillers, microneedling, laser treatments, and chemical peels. Physicians who did not advertise aesthetic treatments were documented with baseline information only. Additional sources such as health platforms or municipal websites were explicitly excluded from the analysis. Physicians with more than one specialty were counted in each corresponding specialty group. If a physician held both a specialty and a general-practice qualification, only the specialty was considered, because in Austria many specialists complete general-practice training during postgraduate rotation, which would otherwise overestimate the number of active general practitioners.

All analyses were performed in Python (v3.14.0). We computed descriptive statistics only, reporting frequencies as absolute counts (n) and proportions (%). Data wrangling used pandas (tabular operations), NumPy (basic computations), openpyxl (Excel I/O), and Matplotlib (figures). Results were exported as tables (CSV/Excel) and graphics.

## Results

**Fig. 1:**
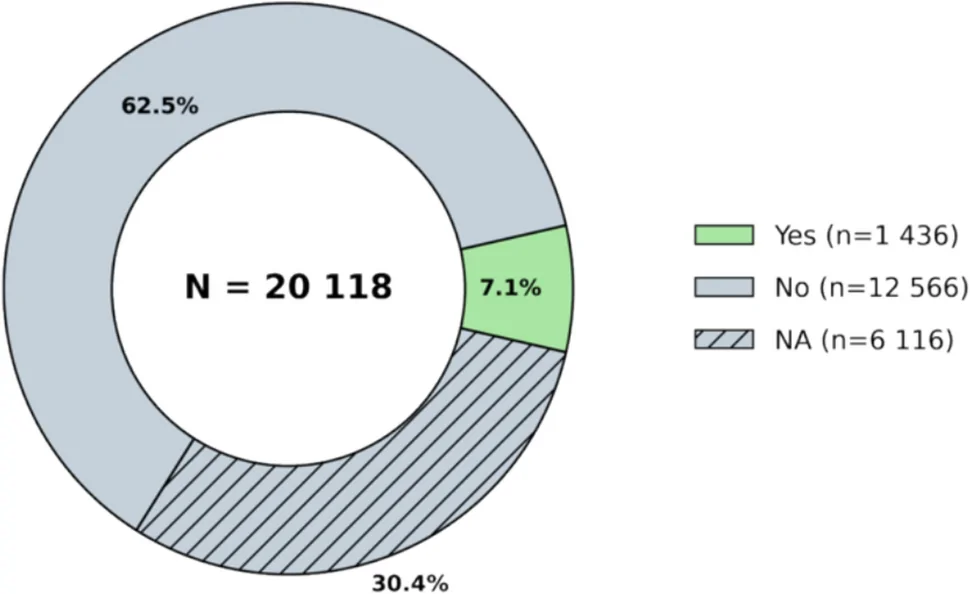
Medical website-based offers of aesthetic procedures (surgical + non-surgical). Yes = list aesthetic services; No = show no reference to aesthetic procedures; NA= website not assessable; N = sample

We identified 19 388 office-based physicians in Austria. After including dual-specialty registrations, counting each specialty affiliation as a separate entry, except for general practitioners (GPs), the analytic dataset comprised 20 118 entries across 32 specialties. Based on practice-website screening, aesthetic services were offered in 7.1% (n=1436). 62.5% (n=12 566) showed no reference to aesthetic services, while 30.4% (n=6116) could not be evaluated due to the absence of a personal website (Fig. 1). Among physicians whose practice websites referenced aesthetic services, 78.9% (1133/1436) could be classified as performing non-surgical aesthetic treatments. In a small subset (n=53), a focus on aesthetic services was referenced, but the specific procedure category (surgical vs. non-surgical) could not be ascertained from the website. Overall, 21 of the 32 examined medical specialties list aesthetic services on their homepages, of which 20 specialties offer non-surgical aesthetic treatments (Table 1).

**Table 1:** Distribution of aesthetic services across medical specialties. Total (n) indicates the total number of physicians within each specialty, * values reflect the number of physicians in each specialty who advertised aesthetic services on their practice homepage, as well as the proportion they represent among all physicians offering aesthetic services and treatments.** specialties not listed in the table did not advertise any aesthetic services. Bold indicates the main representatives

Specialty	Total, n	Aesthetic services (surgical + non-surgical)* (n = 1436), n (%)	Aesthetic treatments (non- surgical)* (n = 1133), n (%)	Aesthetic within specialty (aesthetic services),%	Aesthetic within specialty (non-surgical aesthetic treatments), %
**General Med.**	**6104**	**243 (16.9)**	**230 (20.3)**	**4**	**3.8**
Internal Medicine	2215	11 (0.8)	9 (0.8)	0.5	0.4
Orthopedics	1424	15 (1)	13 (1.1)	1.1	0.9
Gynecology	1374	49 (3.4)	34 (3.0)	3.6	2.5
Psychiatry	1106	1 (0.1)	1 (0.1)	0.1	0.1
General Surgery	887	114 (7.9)	97 (8.6)	12.9	10.9
Ophthalmology	829	203 (14.1)	53 (4.7)	24.5	6.4
Pediatrics	746	1(0.1)	1 (0.1)	0.1	0.1
**Dermatology**	**713**	**387 (26.9)**	**370 (32.6)**	**54.3**	**51.9**
Neurology	702	9 (0.6)	8 (0.7)	1.3	1.1
Trauma Surgery	682	16 (1.1)	15 (1.3)	2.3	2.2
ENT	503	76 (5.3)	29 (2.6)	15.1	5.8
Radiology	467	4 (0.3)	3 (0.3)	0.9	0.6
Anesthesiology	463	19 (1.3)	19 (1.7)	4.1	4.1
Urology	463	2 (0.1)	2 (0.2)	0.4	0.4
**Plastic Surgery**	**249**	**230 (16)**	**204 (18)**	**92.4**	**81.9**
OMFS	182	44 (3.1)	35 (3.1)	24.2	19.2
Physical Medicine	181	4 (0.3)	4 (0.4)	2.2	2.2
Nuclear Medicine	79	4 (0.3)	4 (0.3)	5.1	5.1
Lab. Diagnostics	65	1 (0.1)	/	1.5	-
Cardiac Surgery	39	3 (0.2)	2 (0.2)	7.7	5.1
Others **					

**Fig. 2:**
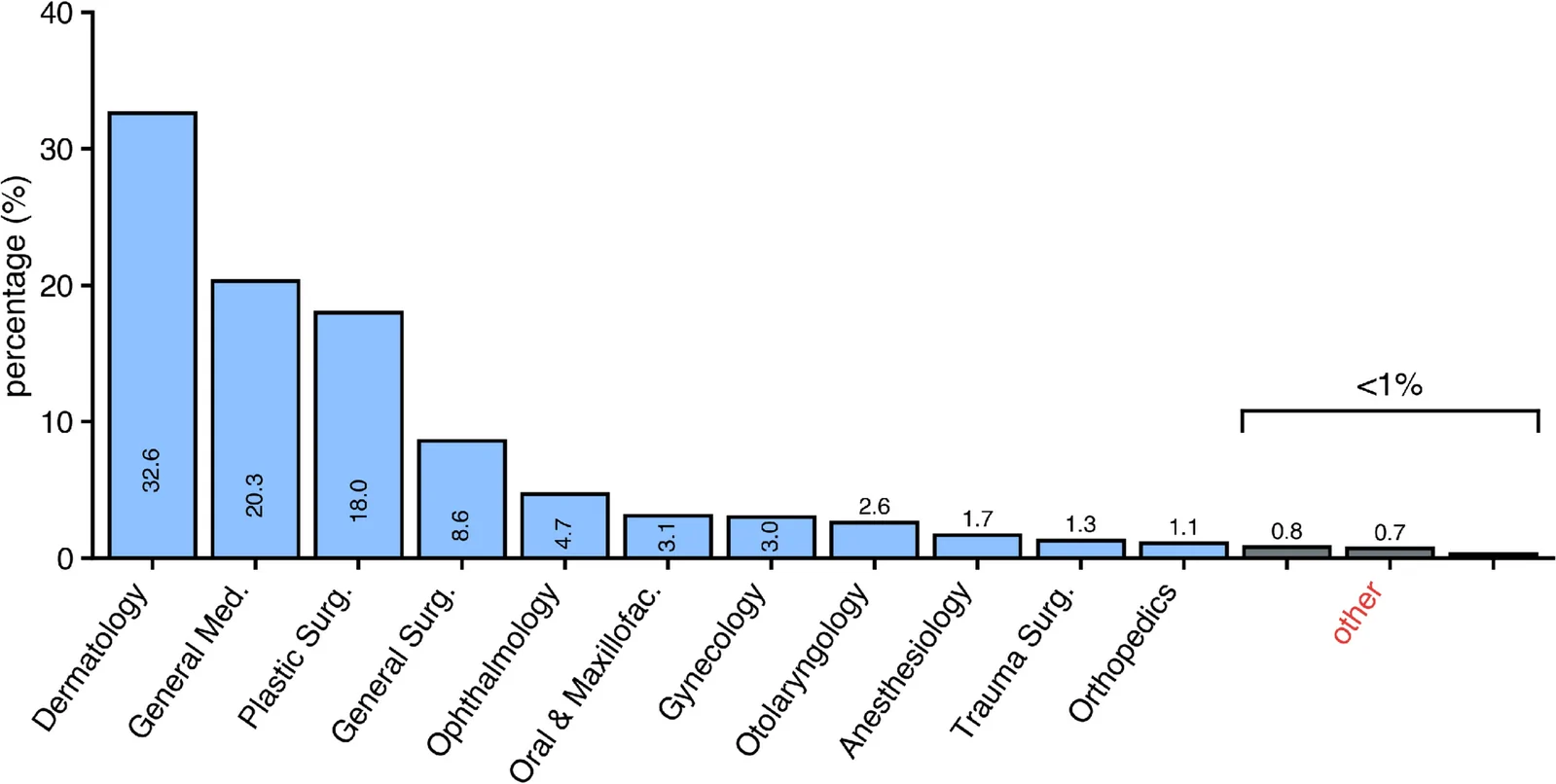
Non-surgical aesthetic treatments (n=1436), sorted by medical specialties

**Fig. 3:**
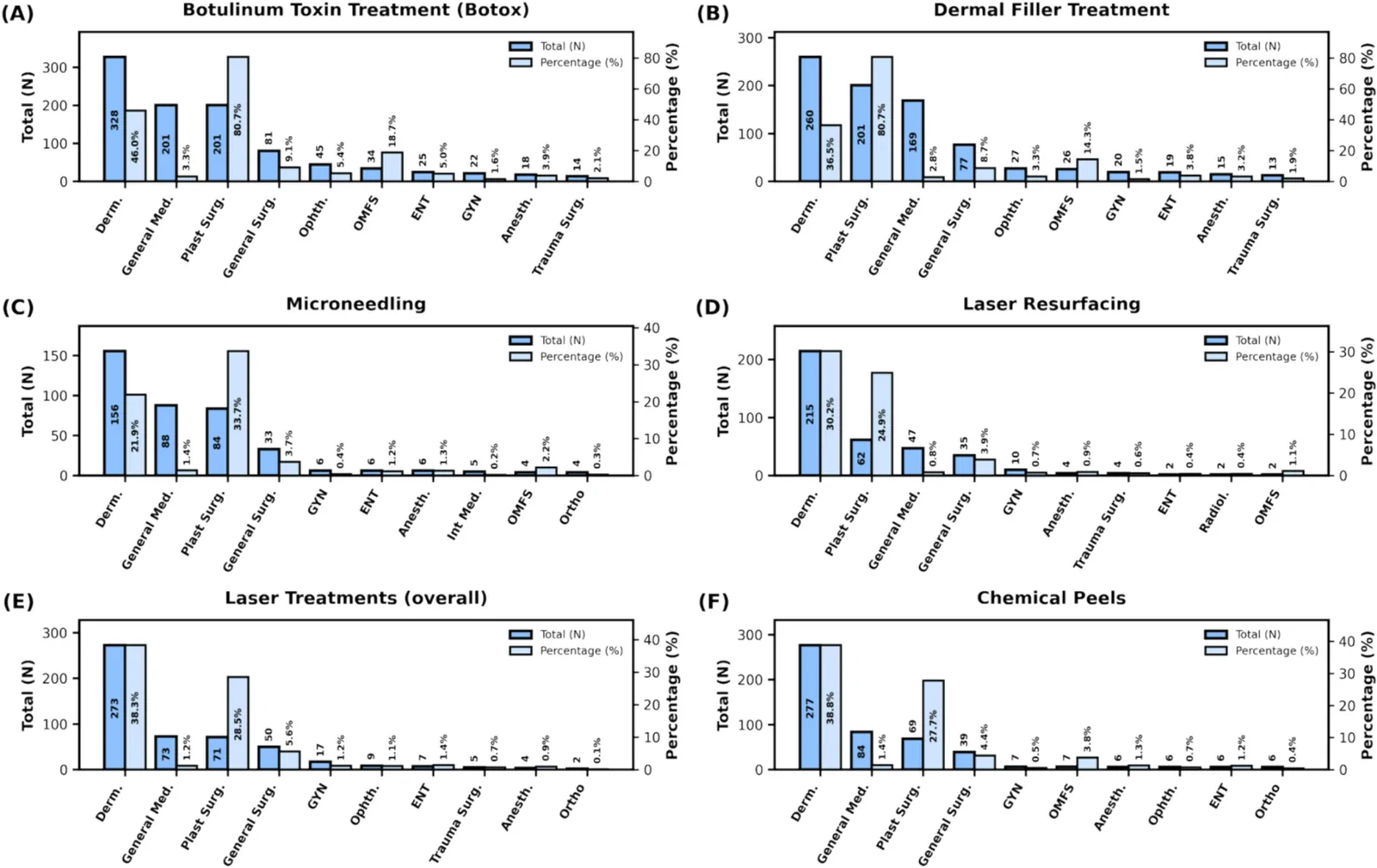
Non-surgical aesthetic treatments. Absolute counts plus percentage within specialty

Non-surgical aesthetic treatments were mostly listed by specialties like dermatologists (n=370; 32.7%), GPs (n=230; 20.3%), and plastic surgeons (n=204; 18%) (Fig. 2). Botulinum toxin and dermal filler treatments were listed on practice websites of all specialties with more than 1% representation within their specialty group. In plastic surgery and dermatology, all examined non-surgical treatment categories were represented, each with a prevalence of more than 20%. Plastic surgeons most frequently listed botulinum toxin injections (n = 201) and dermal filler treatments (n = 201). Dermatologists, however, had the highest absolute numbers across all examined treatment categories, particularly for laser-based procedures and chemical peels. General practitioners (GPs) listed the highest number of botulinum toxin treatments among non-specialty providers (n = 201), ranking behind dermatologists (n = 328), and comparable to plastic surgeons. In absolute terms, botulinum toxin injections were listed by 1012/1133 (89.3%) specialists, dermal filler treatments by 856/1133 (75.6%), microneedling by 406/1133 (35.8%), laser resurfacing by 387/1133 (34.2%), laser treatments overall by 517/1133 (45.6%), and chemical peels by 522/1133 (46.1%) providers. Of all online-listed botulinum toxin treatments, 32.4% were offered by dermatologists, compared with 19.9% by plastic surgeons and a similar proportion by general practitioners (Fig. 3).

**Fig. 4:**
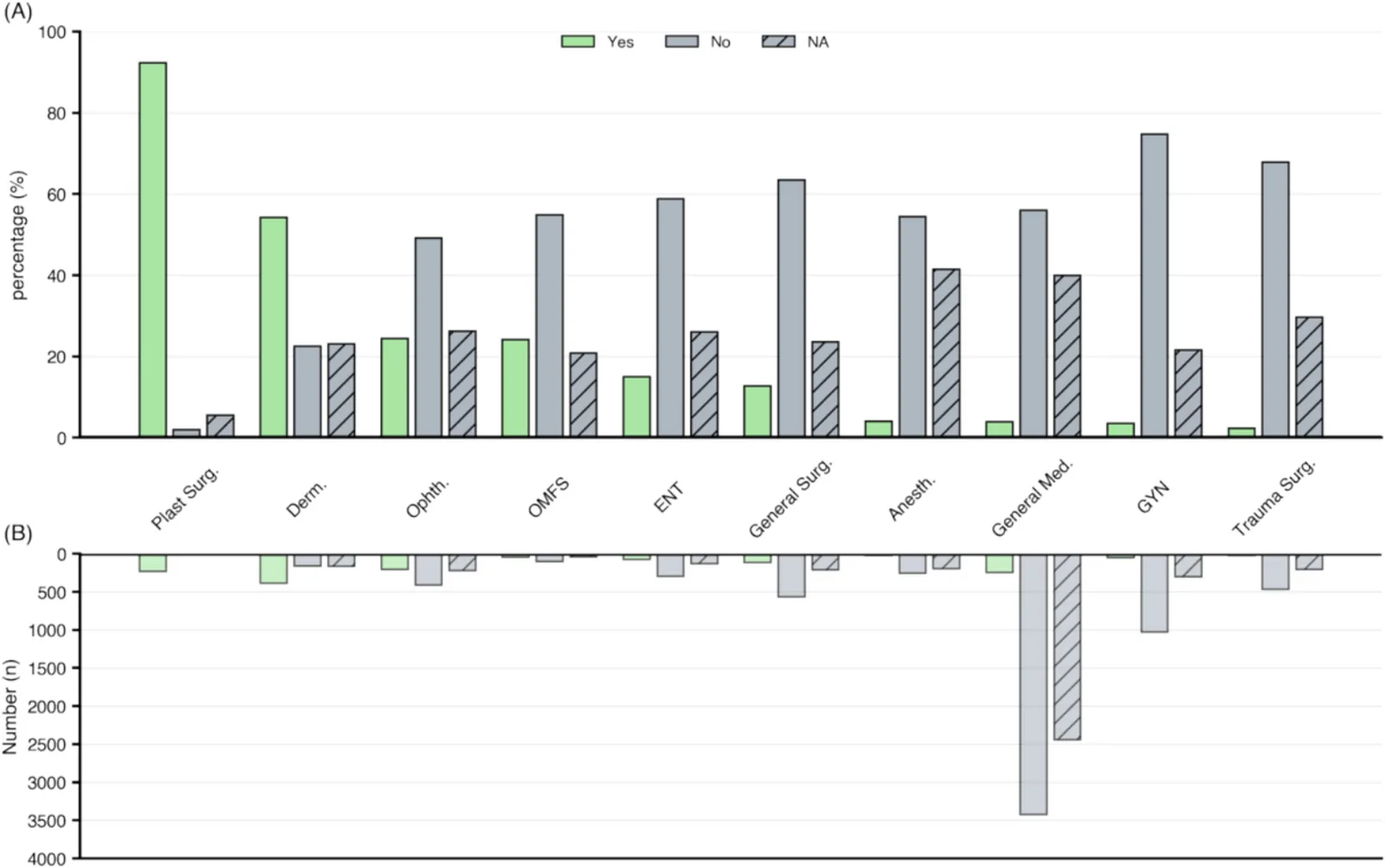
Aesthetic services listed on physician’s practice homepages by medical specialty. (**A**) Percentage within specialty. (**B**) Absolut count within specialty. Yes = list aesthetic services; No = show no reference to aesthetic procedures; NA = website not assessable

Across the specialties as shown in Fig. 4, an average of 25.2% of practice websites was classified as not assessable (NA) regarding whether aesthetic services were offered. This rate was highest for anesthesiologists (41.5%) and GPs (40.0%) and lowest for plastic surgeons (5.6%). Among plastic surgeons, 92.4% explicitly listed aesthetic services; the corresponding proportions were 54.3% for dermatologists, 24.5% for ophthalmologists, 24.2% for oral and maxillofacial surgeons, and 4.0% for GPs (Table 2).

**Table 2:** Aesthetic services listed on physician’s practice homepages by medical specialty. Data are presented as the number of physicians in each specialty and the proportion who: did not list aesthetic services (“no”), listed aesthetic services (“yes”), or whose websites were not assessable (“NA”)

*Specialty*	Total, n	No, n (%)	Yes, n (%)	NA, n (%)
General Med.	6104	3421 (56)	243 (4)	2440 (40)
Orthopedics	1424	1137 (79.8)	15 (1.1)	272 (19.1)
Gynecology	1374	1028 (74.8)	49 (3.6)	297 (21.6)
General Surgery	887	563 (63.5)	114 (12.8)	210 (23.7)
Ophthalmology	829	408 (49.2)	203 (24.5)	218 (26.3)
Dermatology	713	161 (22.6)	387 (54.3)	165 (23.1)
Trauma Surgery	682	463 (67.9)	16 (2.3)	203 (29.8)
Otolaryngology	503	296 (58.9)	76 (15.1)	131 (26)
Anesthesiology	463	252 (54.4)	19 (4.1)	192 (41.5)
Plastic Surgery	249	5 (2)	230 (92.4)	14 (5.6)
OMFS	182	100 (54.9)	44 (24.2)	38 (20.9)

## Discussion

This is the first study in the Central Europe to quantify how often non-surgical aesthetic treatments are advertised on practice websites across medical specialties. For plastic surgery specifically, international detailed public statistics are available through annual society reports and international surveys, covering volumes, temporal trends, and patient characteristics [4, 5, 10]. However, comparable data for other specialties are lacking as reporting is fragmented. Much activity occurs in private or non-hospital settings without mandatory registries. Our website-based study addresses a part of this gap by providing a specialty-based analysis of non-surgical aesthetic treatments in Austria. The novelty of the present study lies in its provider-centered, country-specific dataset. Rather than estimating procedure numbers, this study maps the publicly advertised aesthetic treatment portfolio of office-based physicians across Austria and links these offerings to medical specialties. This approach uniquely complements previously published international surveys by capturing how aesthetic medicine is distributed across disciplines in everyday outpatient practice under a defined legal framework.

We observed a heterogeneous provider landscape, both in terms of the specialties represented among physicians offering aesthetic services on practice websites and the proportion of physicians within each specialty advertising such services. Among all physicians advertising non-surgical services on practice websites, dermatologists represented the largest fraction (32.7%), followed by GPs (20.3%), while plastic surgeons accounted for a smaller proportion (18%). These findings mirror international trends toward diversification of providers. In Southern California, for example, only 26.5% of hyaluronic-acid filler procedures in 2010 were performed by plastic surgeons [11]. Earlier in 2007, D’Amico’s survey states that plastic surgeons were in only 7% the initial consumer choice for minimally invasive procedures, prompting calls for broader non-surgical offerings within plastic surgery to remain competitive [12]. Consistent with this diversification, Zargaran et al. reported that among doctors providing injectable aesthetic treatments in the UK, the largest groups were general practitioners (31.29%), plastic surgeons (25.57%), dermatologists (12.00%), and general surgeons (9.71%) [13]. Taken together, our results complement procedure-based and society-level statistics by revealing how non-surgical treatments are positioned across specialties in Austria’s public-facing online websites.

The broad range of medical specialties offering non-surgical aesthetic treatments, as observed in our study, reflects an increasingly heterogeneous provider landscape. While this variety may enhance accessibility for patients, it also underscores an enduring tension between access and safety, particularly with respect to training standards. Competence and experience are central to satisfactory outcomes and complication management. Plastic surgeons receive comprehensive, anatomy-based training so they are uniquely qualified in their ability to perform aesthetic procedures across all body regions [14]. International data demonstrate that inconsistent qualifications among providers are not uncommon. In the UK, up to 23% of aesthetic procedures are reported to be performed by practitioners without formal medical training [15]. Similar patterns have been described in the USA, where a portion of clinicians offering aesthetic services lack board certification in plastic surgery. Although many providers operate within surgical disciplines, the presence of non-certified practitioners highlights ongoing variability in training pathways [16]. Data from the USA further support these concerns. About 64% of relevant malpractice claims originated in non-medical facilities, with nearly 80% involving medical spas [17]. Burns, pigmentary changes, and other procedure-related injuries were most frequently reported in cases involving non-physician providers. Concordantly, adverse events appear more frequent in non-physician settings, underscoring variable training and the need for clearer safeguards [15, 18].

In Austria, physicians from any specialty may in principle offer non-surgical aesthetic treatments, but advanced training in aesthetics is individualized and not standardized. In our analysis, dermatologists (32.7%) and GPs (20.3%) represented the most frequent specialties offering such services, but several other fields, including gynecology, ophthalmology, otolaryngology, and anesthesiology, also advertise aesthetic treatments on their websites. This raises important questions regarding the extent of anatomical expertise, the variability in non-standardized training pathways, and the potential implications for complication management. It remains unclear whether all providers possess the necessary procedural training and clinical preparedness to adequately recognize and treat treatment-related adverse events.

Across specialties online visibility varied substantially. While almost all plastic surgery practices had functional website that clearly listed their aesthetic services, 40% of general-practice websites could not be evaluated due to absent, outdated, or insufficient online content. Overall, across the main specialties, an average of 25.2% of practice websites were “not assessable” with respect to whether aesthetic services were offered. The uneven web footprint reflects heterogeneity in the public accessible information and suggests that the true prevalence of aesthetic offerings may be underestimated, as non-assessable practices might still provide such services without documenting them online. The true prevalence of aesthetic offerings in these groups may therefore be higher than observed, and differences between specialties should be interpreted with caution.

Our findings align with current social trends. Non-surgical aesthetic treatments are increasingly presented and accepted as routine self-care, part of ongoing body improvement [19]. As views on wellness, beauty, and healthy aging have shifted, people have become more comfortable using aesthetic treatments [20]. ISPAS reports that the most common non-surgical aesthetic treatments are botulinum toxin injections, hyaluronic-acid fillers, hair removal, chemical peels, and non-surgical fat reduction [1]. In our sample, botulinum toxin and hyaluronic-acid services were listed by almost all specialties, especially by dermatologists, plastic surgeons, and GPs, reflecting this convergence of beauty and wellness in everyday clinical marketing.

## Conclusion

In this national, web-based study, 84% of all offered aesthetic services were provided by specialists other than plastic surgery. Non-surgical aesthetic treatments were most frequently listed by dermatologists, followed by GPs. Botulinum toxin and dermal filler treatments were referenced on practice websites of nearly all specialties. These patterns point to a broad, interdisciplinary involvement in aesthetic medicine in Central Europe and should be subject to further investigations.

## Limitations

There are clear limitations to using public websites to map services. Online content may be outdated or incomplete, not all relevant information is publicly accessible, and self-reported descriptions can lead to misclassification and bias. Our design does not capture procedure volumes, practitioner experience, indications, settings, or outcomes. These factors should temper interpretation and motivate linkage studies that connect provider type, training, and practice environment to clinical results.
